# Confirmation of Calcium Phosphate Cement Biodegradation after Jawbone Augmentation around Dental Implants Using Three-Dimensional Visualization and Segmentation Software

**DOI:** 10.3390/ma14227084

**Published:** 2021-11-22

**Authors:** Qusai Alkhasawnah, Sera Elmas, Keywan Sohrabi, Sameh Attia, Sascha Heinemann, Thaqif El Khassawna, Christian Heiss

**Affiliations:** 1 Experimental Trauma Surgery, Faculty of Medicine, Justus-Liebig-University, Aulweg 128, 35392 Giessen, Germany; dr.qusai@jgdc-dental.com (Q.A.); sara.elmas@med.uni-giessen.de (S.E.); christian.heiss@chiru.med.uni-giessen.de (C.H.); 2 Center of Dental Implants, Jordan German Dental Institute, Mamdouh Al Saraireh Street 5, Amman 11821, Jordan; 3 Faculty of Health Sciences, University of Applied Sciences, Wiesenstrasse 14, 35390 Giessen, Germany; keywan.sohrabi@ges.thm.de; 4 Department of Oral and Maxillofacial Surgery, Justus-Liebig University of Giessen, Klinikstr. 33, 35392 Giessen, Germany; sameh.attia@dentist.med.uni-giessen.de; 5 INNOTERE GmbH, Meissner Street 191, 01445 Radebeul, Germany; S.Heinemann@innotere.de; 6 Department of Trauma, Hand and Reconstructive Surgery, Justus-Liebig University Giessen, Rudolf-Buchheim-Street 7, 35392 Giessen, Germany

**Keywords:** calcium phosphate cement, dental implants, immediate dental implants, sinus lift, bone augmentation, dental implants loading

## Abstract

The use of autologous bone graft for oral rehabilitation of bone atrophy is considered the gold standard. However, the available grafts do not allow a fast loading of dental implants, as they require a long healing time before full functionality. Innovative bioactive materials provide an easy-to-use solution to this problem. The current study shows the feasibility of calcium phosphate cement paste (Paste-CPC) in the sinus. Long implants were placed simultaneously with the cement paste, and provisional prosthetics were also mounted in the same sessions. Final prosthetics and the full loading took place within the same week. Furthermore, the study shows for the first time the possibility to monitor not only healing progression using Cone Beam Computer tomography (CBCT) but also material retention, over two years, on a case study example. The segmented images showed a 30% reduction of the cement size and an increased mineralized tissue in the sinus. Mechanical testing was performed qualitatively using reverse torque after insertion and cement solidification to indicate clinical feasibility. Both functional and esthetic satisfaction remain unchanged after one year. This flowable paste encourages the augmentation procedure with less invasive measure through socket of removed implants. However, this limitation can be addressed in future studies.

## 1. Introduction

Bioactive bone grafts promote bone healing and support implanted materials [1,2]. Synthetic bone substitute materials have shown promising results in achieving the exact purpose of biological grafts despite the lack of living cells [3]. The low cost of manufacturing and lower invasiveness caused an ample increase in implanting bone grafts and dental implants. One of the advantages of bone substitute materials is biodegradability, which hints at a better osteointegration and tissue remodeling [4]. However, material retention is proven on the preclinical level as it is hard to confirm in patients, which is crucial for the clinical evaluation of materials.

In present-day dentistry, dental implants are the best choice for teeth replacement. Dental implant healing is defined by primary stability between the jawbone and the implant surface known as osseointegration. The term was first coined by Brånemark [5] and has been a significant scientific breakthrough in dentistry over the past 40 years [6]. Clinically the reverse-torque method is accepted to examine implant stability based on osseointegration [7]. Osseointegration and the long-term success of a dental implant are dependent on sufficient bone quality and volume [6].

One of the common reasons for the decrease of bone volume at the alveolar ridge is teeth loss in the posterior maxilla. In turn osseointegration of dental implants in the low bone volume area is difficult to achieve [8], which may require bone augmentation [9] before dental implant placement.

In severe cases of posterior maxilla atrophy, augmentation of the sinus is the clinical procedure of choice to ensure implant osseointegration [10]. The sinus augmentation was described by Breine and Branemark using a particulate tibia bone graft apically over the implant on the maxillary alveolar ridge. The study reported a 25% improvement of implant osseointegration [11]. Tatum described the lateral window approach for sinus lifting in 1986; it is based on opening a window into the sinus (antrostomy) through the buccal bone [12], arguably for better visualization of the area. Several studies tried to improve bone healing by mixing the platelet-rich plasma (PRP) with autologous iliac crest bone in the sinus lift surgery. However, the short and long-term results were not positive for these procedures [13,14,15]. Additionally, the use of autologous bone graft is related to donor-site morbidities and therefore terrified many patients from the sinus lift surgery [16].

Furthermore, using long implants (>6 mm) was reported to be more successful than using short implants (≤6 mm) in atrophic regions [17]. Short implants (≤6 mm) were found to have lower predictability in survival rates compared to longer implants (>6 mm) after periods of 1–5 years in function [17]. Lemos et al. included both posterior maxillae and mandibles in their meta-analysis and found that short implants exhibited a greater risk of failure compared with longer implants [18]. Furthermore, Nisand et al. reported similar findings when comparing outcomes for the posterior maxilla and mandible and the options of vertical GBR combined with longer implants vs. short implants [19]. Based on recent findings, increasing dental implant length is considered to play a fundamental role in increasing dental implant primary stability, even with poor bone quality, through controlling the bone preparation process [20], hence the growing need for vertical augmentation as reports estimated that up to 50% of all dental implant procedures currently performed involve the use of bone grafts [7].

Preoperative assessment of such periprosthetic surgery is crucial to its success [21]. When the volume of bone between the ridge crest and the maxillary sinus floor is less than 5 mm, it is considered inadequate, and an open Sinus lift procedure is required [22]. Open SLP allows oral surgeons to elevate the sinus floor to the necessary extent, i.e., vertical augmentation. During SLP, bone graft material is introduced into the sinus and placed beneath the elevated sinus membrane. This makes the sinus lifting for implant placement one of the most demanding procedures for suitable bone grafts, and therefore, this procedure was chosen in this study.

However, bone graft materials vary between biological and synthetic, and the choice of materials is recommended according to the size and topography of missing or defective bone [23]. Synthetic biomaterials have the advantage of low cost and continuous availability and different formulations which have proven effective, especially in diseased bone as in osteoporosis [24].

Nonetheless, since the availability of cone-beam computed tomography scan (CBCT) [25,26] such imaging is recommended for pre-operative assessment of the maxillary sinus. Due to the size of the defect and the strenuous surgery, the use of suitable synthetic bone substitute materials in the sinus is advantageous. Although Calcium phosphate cement (CPC) is the most common bioactive and biodegradable biomaterial extensively studied in the repair of bone defects [27,28,29,30,31,32,33], it was not available in injectable form until recently [34,35]. CPC was reported successful in vertical augmentation of the maxillary sinus floor and hydroxyapatite-coated dental implants [36]. However, when using autografts or CPC, it requires a period of six to nine months until healing is complete before implant placement or loading [37].

In the health sector, cost is a crucial determinant. Globally, current statistics indicate that approximately 2.2 million bone graft procedures, costing an estimated USD 664 million by 2021, are being performed each year, with the number of operative procedures for repairing bony defects estimated to grow by approximately 13% annually [8]. As of 2018, the market value for dental bone substitutes has been estimated to be worth approximately USD 493 million and is projected to grow to approximately USD 931 million by 2025, at a combined annual growth rate of 9.5% [9]. Despite this widespread use of bone graft and substitute materials globally, there are still limitations that remain associated with currently used materials and the clinical evaluation regarding their biodegradability.

The limitations primarily involve the use of allografts, the transfer of grafting materials between two genetically unrelated subjects, and autografts, the transfer of grafting material from one body site to another within the same subject [2]. To our knowledge, none of biological products in the market currently possesses all the ideal properties for a bone substitute material including low patient morbidity, ease of handling, low immunogenicity, low cost and angiogenic potential [10,11,12]. The disadvantages of autografts include the lack of availability of graft tissue, associated pain, morbidity at the donor site and the need for two operative procedures. In contrast, disadvantages of allografts include rejection of the donor tissue by the recipient’s immune system and concerns with transmission of diseases, such as HIV and hepatitis [8,13]. However, their advantage is the almost guaranteed biodegradability.

In recent years, there has been an increased drive in the market to use newer bone grafting materials, such as bone substitute products, despite little evidence-based research for indications and safety [10]. Thus, these matters of concern, along with continued marked increases in demand for bone graft materials and the global ageing population strongly indicate a need for further research into the development of novel materials used for bone grafting procedures [8,9,12].

The need for innovative bone substitute material to address this common problem can offer a promising solution for oral surgeons. The prerequisites of such cement are its stability and biodegradability, which allow faster implant loading and prompt replacement with bone, not affecting the implant integrity. Therefore, we here examined the feasibility and degradability of a novel calcium phosphate cement paste (Paste-CPC, INNOTERE Germany) as a bone grafting material. Using the conventional window approach, the study utilized the cement paste to perform sinus lift without any additional materials (NO membrane, NO tags and NO pins). This study aimed to assess the reliability of performing immediate loading on novel bone cement simultaneous surgery, with immediately inserted longer dental implants for sinus-lifting, grafting and implant placement in situations of limited residual bone heights (<4 mm) in the posterior maxilla, and to bring a new concept in the loading time on implants by reducing this frame of time from 6 to 9 months down to 8 weeks. Moreover, the study introduced a novel approach for the clinical evaluation of biodegradable bone cement using 3D segmentation within 12 months on a single patient case.

## 2. Materials and Methods

The window approach was used to examine the feasibility of the use of Paste-CPC (INNOTERE GmbH, Dresden, Germany, composition in Table 1) in the sinus. Dental implants with 3.8 mm in diameter and 10.5 mm in length were used (BioHorizons^®^ Tapered Internal implants (BioHorizons, Birmingham, AL, USA). The patient signed an informed consent module before the surgery and all possible outcomes have been explained to the patient.

**Figure 1:**
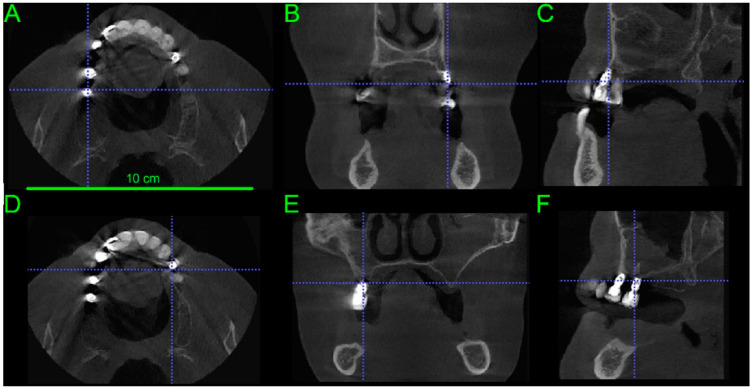
Bone quality determined pre-operatively using CBCT images. Orthogonal views were acquired by using 2D basis images for secondary reconstruction of axial (**A**,**D**), coronal (**B**,**E**) and sagittal (**C**,**F**) views. Significant buccal bone loss is apparent on the left side (upper panel), and the right side (lower panel).

The patient (58 years old female) was diagnosed clinically with bone loss in the dorsal part of the right maxilla using Cone Beam Computed Tomography (CBCT) imaging Vatech, Orangedental, Seoul, Korea). Backward planning was performed to determine possible positions for the replacement implants and the quality and volume of the remaining bone. The bone assessment was done using CBCT to acquire 2D images (Figure 1) using Pax-I software (Oragedental, Seoul, Korea).

**Figure 2:**
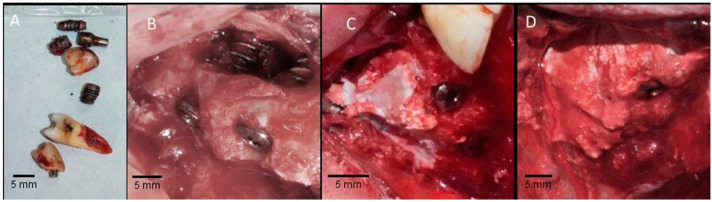
The clinical procedure used in the present case to increase the volume of the buccal bone in the whole area planned for placing the new implants. (**A**) Old implants were removed; (**B**) placement of new implants showing the sinus window; (**C**,**D**) Paste-CPC was used as a bone graft to close the sinus window, filling it up to cover the implants and the missing buccal plate in the mesial implant and flap after healing.

The surgery started at the right quadrant, and the incision was made at the alveolar crest and after the subperiosteal detachment. The flap was raised using a periosteal elevator and attached to the cheek with 4-0 nylon sutures (Ethicon, Johnson & Johnson, Somerville, NJ, USA) to gain access to the maxillary sinus with a spherical diamond drill, taking care not to tear any tissue or the sinus membrane, and the old implants were removed (Figure 2A) using upper posterior forceps. Further, a window in the lateral wall of the sinus was prepared, and tissue curettage was performed. Appropriate elevators were used to detach the sinus membrane, creating the area for the bone graft, and then, drilling for three implants was performed; two implants were inserted in the sinus area and the third in the buccal cortical missing bone area with 3.8 mm in diameter and 10.5 mm in length. Subsequently, the Paste-CPC bone graft was injected into the sinus area to cover the implants and the window and to fill the missing buccal plate in the mesial implant, increasing at the same time the volume on the buccal bone in the whole area (Figure 2C,D).

**Figure 3:**
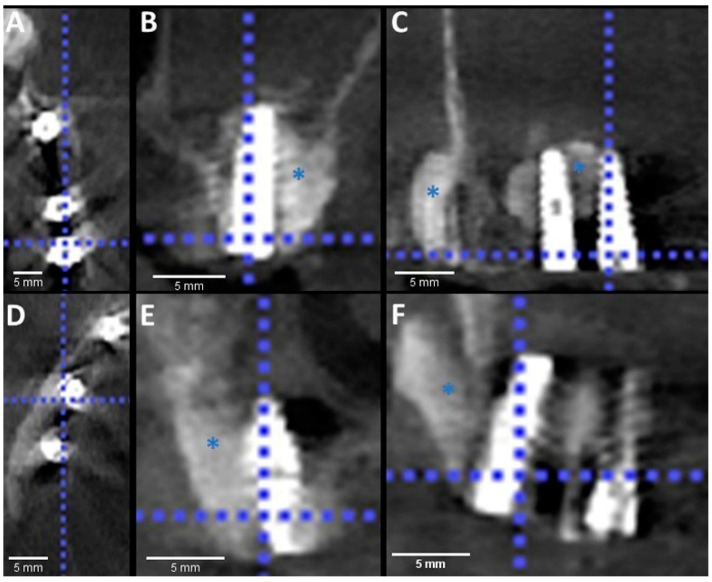
Postoperative CBCT imaging showed good integration between the cement, implant and bone. Paste-CPC is seen around the implants within the bone defect. Zoom enhanced axial (**A**,**D**), coronal (**B**,**E**) and sagittal (**C**,**F**) views, on the left side (upper panel) and the right side (lower panel). Blue asterisks mark the cement.

After 10 min the Paste-CPC material started to harden. Using Nylon stitches 4.0 and continuous matrix technique, the mucoperiosteal flap was realigned and sutured directly over the Paste-CPC material. No membrane was used to cover the facial defect in the sinus wall. Furthermore, the same procedure was performed at the left quadrant. Whereas the second premolar was extracted because of the cystic legion and the removal of an old implant that replaced tooth number 4. A post-operative CBCT scan was performed to visualize the cement placement (Figure 3).

The postoperative procedure was that the patient received Dalacin 600 mg (Pfizer, Berlin, Germany) to manage infection, and SERODASE 10 mg (Kusum, Delhi, India) for seven days to manage inflammation and pain. Further pain management was carried out using Ventor^®^ Nimesulide 100 mg (ReplekFarm, Skopje, Macedonia) if needed, and it was prescribed up to 4 times a day for 7 days. The patient was also provided with a provisional prosthesis in the same session. The patient was advised to avoid hard food during the first weeks to reduce pressure forces at the bone-implant site. In the healing period and the follow-up visits, CBCT was done showing no complication or losses in bone particles. The areas were opened again eight weeks after surgery, and augmentation, screw-retained abutments were inserted with protective caps.

**Figure 4:**
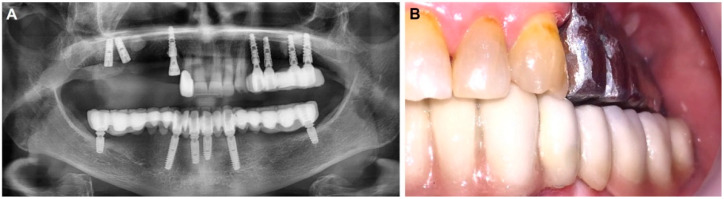
Provisional prosthesis was mounted in the same session after sinus lifting using Paste-CPC. The prosthetic stage started on the tenth week. (**A**) Orthopantomagram shows the metal prosthesis on the right side, and the cement is seen near the cranial end of the implants. (**B**) The metal framework try-in on the right side.

After one week of abutment loading, the patient came again for the prosthetic stage (Figure 4); ten days later we performed and delivered connected bridges of porcelain fused to metal for the patient on both sides using the screw-retained protocol. The patient was recalled for follow-up evaluations one week; eight weeks; and three, six and twelve months after implant placement. During each follow-up visit, a clinical assessment of the implant, peri-implant tissues and the prosthesis was carried out. A comprehensive scenario of treatment is shown in Supplementary Figure S1.

One central claim of bioactive bone cements is biodegradability and bone formation enhancement. Therefore, this article focuses on segmenting CBCT images of the jaw and using 3D slicer software to segment the cement, bone and dental implants to calculate cement volume and retention in time.

3D slicer is a free open-source software developed to analyze medical imaging through visualization and segmentation [38]. The CBCT data sets were delivered as DICOM (Day 1, (months = M) 3 M, 6 M and 12 M) and analyzed using the threshold segmentation method, where the volume of the cement was quantified. The measurement was semi-automated and was replicated three times.

To establish a threshold range tow, trained operators were asked to double blindly select reference values to identify the upper greyscale range (hard tissue) and lower greyscale range (soft tissue); in each time point a CBCT scan was performed, and those two values are then taken from the grey value histogram. The cement was taken within the visually selected area below the grey threshold of hard tissue and above the soft tissue value.

To render the volumes, the segment statistics application of Quantification-module was used. 3D slicer automatically calculates a table with measurements of each segment, after choosing the wanted scalar volumes.

## 3. Results

**Figure 5:**
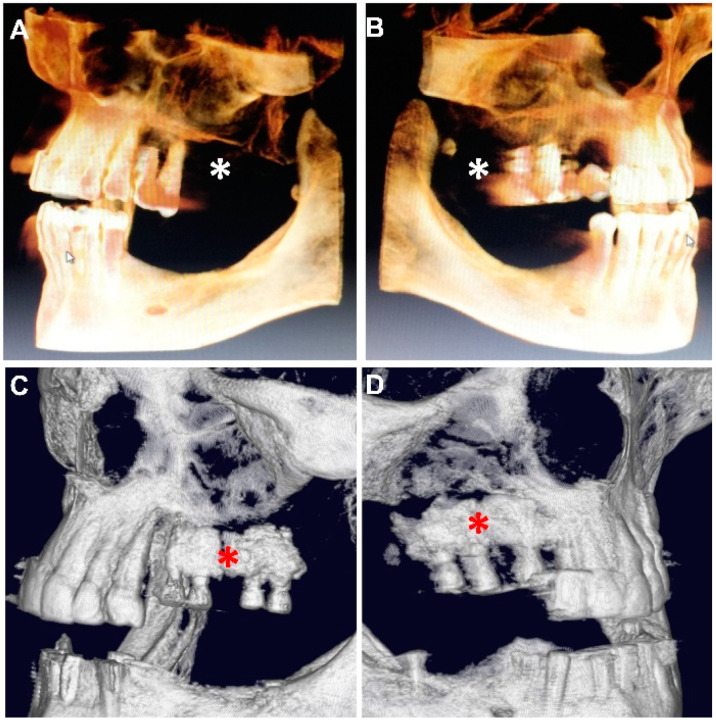
Bone loss and Paste-CPC-filled defect were visualized using 3D reconstruction resulting from CBCT imaging. The large defect area (white asterisks) resulting from significant loss is of the buccal bone on both sides. Bone substitute material surrounds the new implants after injection (red asterisks) using the window approach ((**A**,**C**): left side, (**B**,**D**): right side).

3D reconstructed images using commercial software (Pax-i, Oragedental, Seoul, Korea) from the DIOCM data acquired by the CBCT device (Vatech, Orangedental, Seoul, South Korea) showed bone loss in the dorsal part of the right maxilla. The patient had two short implants (5 mm in length and 4 mm in diameter) at the first and second molar positions and one short and mobile implant in the left quadrant with 7 mm in length and 4 mm in diameter as well as a significant buccal bone loss on the area of fours (first premolars). The 3D image is used to assess the width of each implant and the thickness and density of the cortical plates and cancellous bone (Figure 5A,B). Furthermore, implant loosening was assessed as mobility grade 3 (>2 mm or depressibility in the socket).

Nonetheless, the bone substitute material reflected adequate injectability and easy handling and better usability as it did not harden rapidly nor did it retarded heat. The flowability of the cement allowed it to engulf the implant although the injection was from one direction and after implant placement (Figure 5C,D).

**Figure 6:**
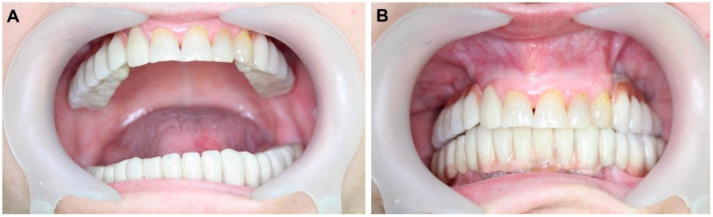
Final prosthesis, ten weeks post-surgery. (**A**) The view with the open mouth shows occlusion. (**B**) The closed mouth view shows the superstructure, vertical dimension and the smile line.

Postoperatively, the implants could withstand a reverse torque of 40 N/cm^2^ as a secondary stability test. This speaks to the stability provided by the hardened Paste-CPC after 30 min. This encouraged the oral surgeon to immediately mount the provisional prosthesis (Figure 4), which was replaced after ten months at the estimated time point of total functional loading (Figure 6).

**Figure 7:**
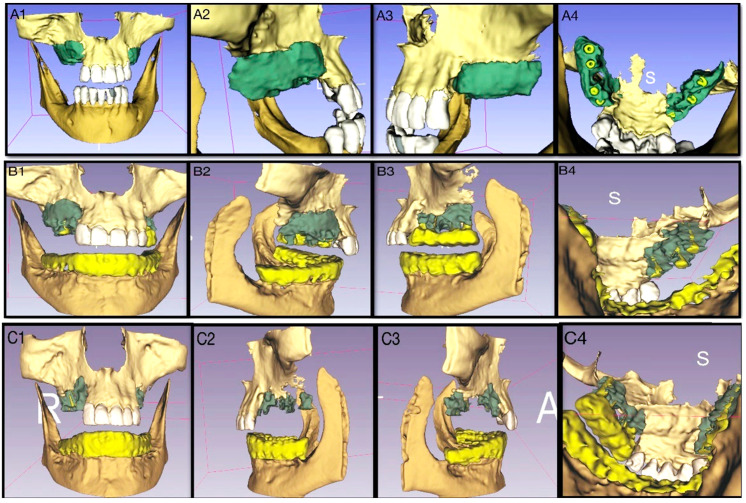
3D visualization and segmentation of upper and lower Jaws. 3D-slicer was implemented to segment the maxillary bone after sinus lift and horizontal augmentation using Paste-CPC at Day 1 and 6 and 12 months postoperatively. Preparation with 3D slicer (Green: Paste-CPC, neon yellow: screw implants, beige-yellow: Jawbone; 1: frontal, 2: left-lateral, 3: right-lateral, 4: palatal). (**A1**–**A4**) shows Paste-CPC placement postoperatively (Day 1). The cement surrounds almost the complete length of the implant on both sides. (**B1**–**B4**): 3D segmentation at 6 M after surgery, visibly reduced bone cement substance compared to Day 1, bone tissue replacing the resorbed areas at the superior cranial border of the bone cement, as well as resorption batches at the inferior wall. (**C1**–**C4**): segmentation at 12 M postoperatively compared to Day 1, and 6 M increased bone cement degradation and resorption.

Segmentation was performed on retrospective follow-up data to compare material retention, bone cement placement and osteointegration. Descriptively, at Day 1, the bone cement was distributed around the implants to a high extent (Figure 7A). However, a small gap of 2.8 mm in diameter could be seen behind the second implant on the left side (Figure 7(A4)), whereas the right side shows a more compact structure of the paste. The degradation in the cement volume was noticeable after the surgery by 26.5% at 3M and by 59.6% at 6 M (Figure 7B). The reduction was highest after 12M by 81.1% (Figure 7C).

**Figure 8:**
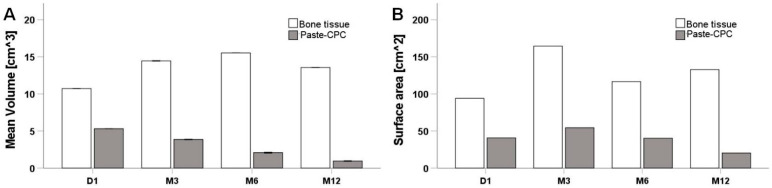
Degradation of bone cement is evaluated in the jaw. (**A**) Paste-CPC volume was reduced with the progression of the healing. (**B**) Surface area did not reflect the degradation of the material until M12.

Therefore, both the volume and the surface area of the cement were evaluated and compared between time points. The bone substitute material’s Paste-CPC volume reflected a study decrease with the healing progression (Figure 8A). However, the bone volume increased at its maximum by M6 before it was intriguingly slightly reduced by M12. The measurement of the cement surface area was, however, fascinating. The Paste-CPC area seemed to increase marginally compared to D1. However, no significant change was seen until M12, with an apparent reduction in the surface area.

## 4. Discussion

The rehabilitation of partially or edentulous patients with implant-supported prostheses has become common practice in dentistry; however, the posterior maxilla frequently represents a challenge because of the lack of bone due to alveolar ridge resorption and maxillary sinus pneumatization. A lateral sinus lift is one of the most used augmentation procedures, it enables to make an implant in the dorsal parts of the maxilla, where the bone often has poor quality and is reduced by the extended maxillary sinus [29]. When considering that the minimum safe length of the implant is 10 mm [30], it was found that in 25% of patients, the level of the bone is considered very low at the site of the first premolar, whereas 50% of patients have an insufficient level of bone at the level of the second premolar, increasing up to 80–90% at the level of molars. Thus, the purpose of this study was, on the one hand, to address the feasibility of using a novel pasty calcium phosphate cement (Paste-CPC) as augmentation material for dental implants to enhance the healing process and enable immediate or early loading. On the other hand, the study provides the first evidence of the possibility of measuring the degradation of bioactive bone cement in the patient from retrospective 3D imaging sources such as CBCT DICOM data set open-source software.

In the present study, similarly to other studies, no membrane was used, thus reducing the costs of surgical procedure, even though it is expected that they prevent bone resorption by maintaining space and secluding the grafted area from connective tissue. However, there is continuing debate regarding whether membranes should cover the augmented site [31].

The results indicated that the implementation of Paste-CPC is encouraging; on the one hand, it is an injectable paste which makes the handling very easy, and on the other hand, it enables simultaneous surgery for sinus lifting and grafting. Dental implants in the posterior maxilla with shallow vertical depth can be performed safely. In addition, they reduce the number of surgical interventions, time and costs [25,32]. Paste-CPC showed low setting time which reduces the implant disintegration. Moreover, no additional materials were needed, and the time loading was extremely reduced by using Paste-CPC from nine months to eight weeks. Of course, other protocols proposed to reduce the time before loading to a few weeks successfully. Many of these techniques were based on optimizing bone density to improve primary stability, such as placing the implants 1–2 mm subcrestally [39,40]. Other techniques depend on bicortical fixation of the implant into the nasal or sinus floor [41] or using osteotome to condense bone [42]. Another common technique is implant site preparation where the final drill used has a smaller diameter than the diameter of the implant [43]. However, unlike the current study, most articles on immediate or early loading do not use bone graft of sinus lift procedures and recommend conventional loading [39,44,45,46,47,48].

Furthermore, we showed the possibility of introducing 3D visualization and segmentation to address the integration between the cement and bone as well as the degradability of the cement. The latter is usually promised by the manufacturer based on preclinical studies where the animal models are sacrificed, and histological analysis occurs.

Furthermore, Contemporary literature also showed that performing a single step for the sinus floor elevation, grafting and dental implant placement has a high success rate [26,32,33]. Bioactive materials such as Paste-CPC, a ready-to-use bone grafting material, could bring a new treatment rule besides the golden rule of Tarnow [49] that says “no buccal bone, no implant”, or the six months waiting is a must in the sinus lifting procedure.

Furthermore, bioactive, biocompatible and biodegradable biomaterials are favorable for replacing missing bone with no risk of immunogenicity or toxicity. Moreover, biomaterials can provide immediate stability to the implant and the operated region [50]. Although autogenic and allogeneic bone grafting are biologically superior to synthetic biomaterials, poor quality and limited quantity of bone in elderly patients, multiple operation sites in one patient or the donor-to-donor immunogenicity limit the use of biological materials.

One of the most common bioactive and biodegradable biomaterials extensively studied in the repair of bone defects, in neurosurgery, orthopedics trauma, plastic surgery and dentistry [27,28,29,30,31,32,33] is calcium phosphate cement (CPC). Despite the rapid development in biomaterials, suitable injectable synthetic materials to substitute bone were not available until recently [34,35]. A CPC paste is more common in the form of powder and liquid, which when mixed sets as primary hydroxyapatite. Grafting applications of CPC paste are more common on trauma and orthopedics than in the dental community [49]. However, studies report that partial or full healing is required before implant placement or loading [36,37].

The need of innovative bone substitute material to address this common problem can offer a promising solution for oral surgeons. The prerequisites of such cement are its stability and biodegradability, which will allow a faster loading of implant and be replaced with bone in a timely manner not affecting the implant integrity. Those properties are found in the Paste-CPC.

This material has been extensively investigated due to its excellent biological properties, potential biodegradability, molding capabilities, and easy handling. Because the material can potentially be replaced by bone after a while, it could retain the short-term biological advantages of hydroxyapatite without the long-term disadvantages. Further innovations can also be reached through doping the cement with further bone formation molecules. For example, Thormann et al. revealed that strontium doped Paste-CPC shows enhanced bone formation due to the release of Strontium [35].

This literature review reports on the dental bone graft and substitute materials that are currently available commercially, their limitations and the potential development of promising alternatives brought about by the emergence of synthetic bone substitutes in recent decades.

The maxillary sinus consists of more cortical bone than cancellous bone on the buccal side. This means less vascularization and low number of osteoprogenitor cells, and it affects the capacity to form new bone [51]. Therefore, reconstruction of maxillary sinuses larger than 2 cm^3^ is recommended [52] and should be considered critical defects where augmentation is the best choice [53].

The protocol suggested in this study contributed to the reported biomaterials degradation. The performed area vascularization brings the biomaterials in contact with body fluids leading to trigger the biologic response [54] (Figure 2, Figure 7 and Figure 8).

Such contact works chemically and biologically to aid biomaterials degradation. Furthermore, CPC is considered an osteoconductive material which can enhance cell migration into the formed scaffold [55]. This enhances the benefit of the low number of cells around the scaffold and promotes material degradation and bone formation simultaneously. Furthermore, bone formation and material degradation are positively affected by mechanical stimulation which was ensured through the early implant loading [56].

Preclinical studies on the Paste-CPC utilized in this study reflected the enhancement of bone healing and increased degradation compared to empty defect in rat model through histological, chemical and mechanical examination [50,57,58]. Those results are in line with this study as reflected in the volume measurements (Figure 8)

One major limitation is the proof of biodegradability in living patients due to the limited possibility and technology; however, we could show in this study that segmentation using 3D slicer is possible and reliable. Nonetheless, the 3D slicer software depends on experienced operators to define the grey value range in contrast to other research software such Materialise Mimics Software which requires the use of a fantom to identify the grey values. Indeed, the subjectively set greyscale ranges can influence the results. However, only the area of defect was measured, and batches with the maximum greyscale value were considered as new bone formation. This led to the exclusion of tissue that is not fully mineralized to avoid misleading results. Therefore, the change in the bone volume was not contrasting in pattern with the material degradation. Therefore, the presented segmentation method is more robust when investigating biomaterial degradation rather than bone formation. Nonetheless, the authors are currently working on comparing the results of both software packages; however, we see the value of using opensource software in that it encourages researchers to evaluate retrospective data when the possibility to run a fantom on the same day of the scan has passed.

The limitation of our proof-of-concept study is the low number of examined implants and regions. However, such studies will pave the way for a larger study using the cement and the 3D segmentation software. Further studies are planned with 15 patients for a minimally invasive approach for sinus lifting. Later, we propose a study that compares the Paste-CPC with a control under a valid statistical power.

The use of such flowable biodegradable paste can reflect on the SLP, making it less invasive and moving from the window approach to injecting the paste through the implant socket before new implant placement.

## 5. Conclusions

The consequences of using hydrophobic calcium phosphate cement (Paste-CPC) have significant implications for dentistry. The proof of its biodegradability in patients shall help in further investigations of bone cement in dentistry. Furthermore, a less invasive surgical procedure with reduced implant–prosthetic rehabilitation time and cost of surgery can be developed and approved as a suitable method. The outcomes obtained from the present study provide a safe and easy method of small surgery for younger or less experienced dentists. The findings of this study will be helpful as a resource material for dentists, researchers, scientists and universities. However, further prospective randomized studies for more improvement are required.

## Figures and Tables

**Table 1:** Composition of INNOTERE Paste-CPC.

Components with Percentage (%)	
alpha-tricalcium phosphate	48.35–49.88
calcium hydrogen phosphate (monetite)	20.95–21.61
calcium carbonate (calcite)	8.06–8.32
tricalcium ortho-phosphate (CDHA)	3.23–3.33
dipotassium hydrogen phosphate	2.41–2.49
Miglyol 812 (caprylic/capric triglycerides)	11.56–13.68
Kolliphor ELP (poly-oxyl-35-castor oil)	2.11–2.50
Amphisol A (cetyl phosphate)	0.70–0.82

## Data Availability

The data presented in this study are available on request from the corresponding author.

## Supplementary Materials

The following are available online at https://www.mdpi.com/article/10.3390/ma14227084/s1, Figure S1: The clinical scenario for the represented case treatment performed at the Center of Dental Implants, Jordan German Dental Institute (JGDI). (A) Pre operative radiographic view acquired by using 2D basis images for Secondary reconstruction from the CBCT slices, The view shows the short implants on both sides of the upper jaw. (B) clinical photograph for the pre operative condition. (C) missing prosthetic part of the implant and adjacent crown showed mobility due to bone loss (poor prognosis) on the right side of the upper jaw. (D) the exposed short implants after removing the prosthetic part. (E) short implants were removed from the right side of the upper jaw. (F) after flap reflection the available bone shows sever loss (G) placement of new implants and showing the sinus window; (H) Paste-CPC was used as a bone graft to close the sinus window, and filled up the cover the implants and the missing buccal plate in the mesial implant and flap after healing. (I) removal of prosthetic part from the left side of the upper jaw. (J) Flap opening. (K) bone loss is also apparent on the left side. (L) placement of new implants on the left side; (M) Paste-CPC was used as a bone graft to close the sinus window, and filled up the cover the implants. (N) Radiographic image (OPG) represents the inserted long implants and the injected cement. (O) suturing of the flap. (P) wound healing after 10 days of surgery. (Q) Gingival formers were placed for tissue management. (R) Impression posts were used for indirect backup impression technique for the upper jaw. (S) Screw retained metal framework Try-in on both sides. (T) screw retained suprastructure at week 10 post surgery. (U) Radiographic view acquired by using 2D basis images for Secondary reconstruction from the CBCT slices, The view showing the inserted implants on the lower jaw after teeth extraction and upper jaw after sinus lifting. (V) Impression posts were used for indirect backup impression technique for the lower jaw. (W) Screw retained metal framework Try-in for the lower jaw. (X,Y) final result showing the permanent prosthetic parts on the lower and upper jaw, reflecting the resulted finish line and the occlusion.
