# Supplementary material for: Confirmation of Calcium Phosphate Cement Biodegradation after Jawbone Augmentation around Dental Implants Using Three-Dimensional Visualization and Segmentation Software
Source: Materials (Basel). 2021 Nov 22;14(22):7084. doi: 10.3390/ma14227084 (PMC8618138; doi:10.3390/ma14227084)
Supplement: Supplementary file 1 [file materials-14-07084-s001.zip › materials-1431801-supplementary.pdf]

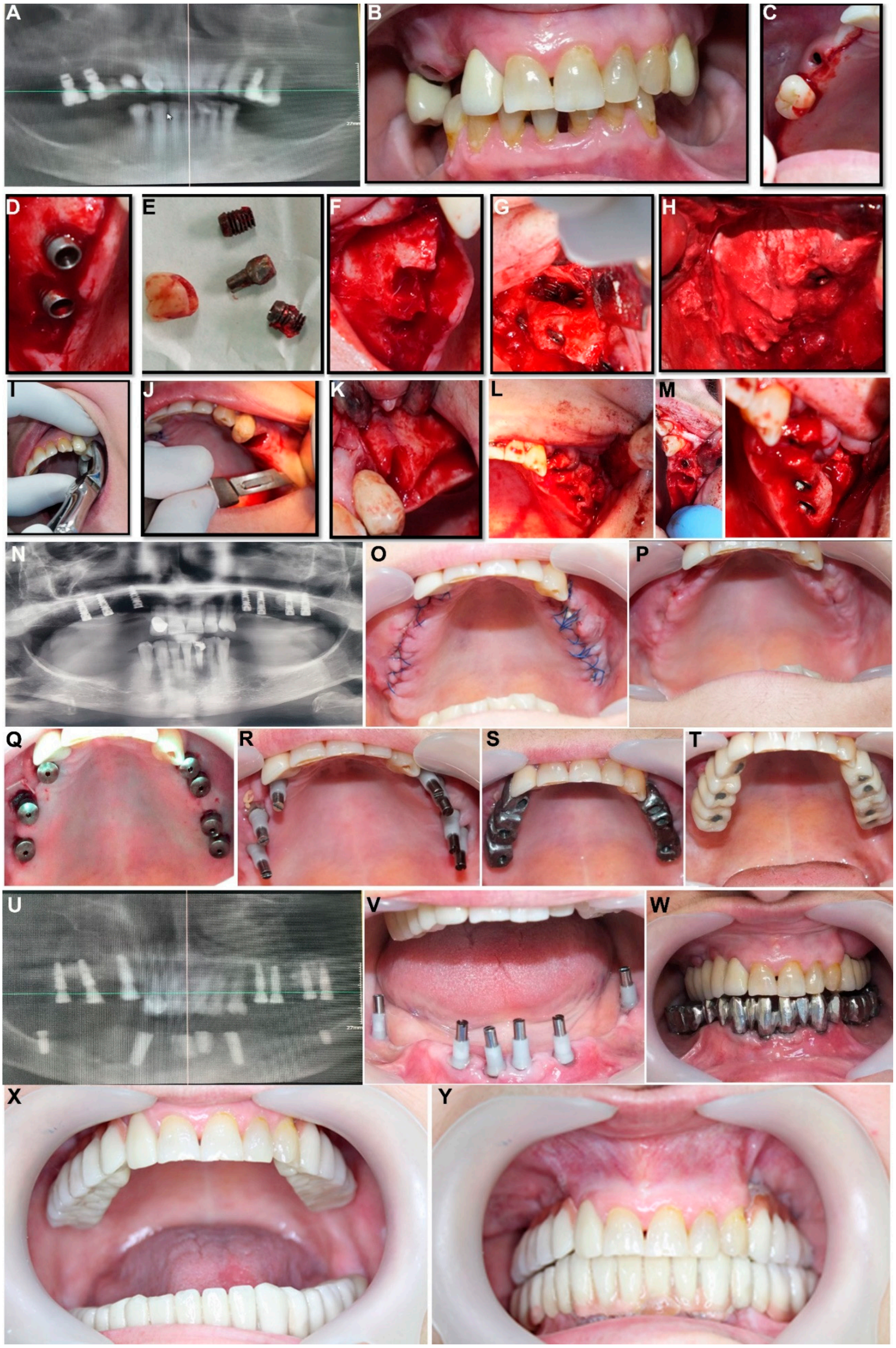
A
B
C
D
E
F
G
H
I
J
K
L
M
N
O
P
Q
R
S
T
U
V
W
X
Y

Figure S1. The clinical scenario for the represented case treatment performed at the Center of Dental Implants, Jordan German Dental Institute (JGDI). (**A**) Pre operative radiographic view acquired by using 2D basis images for Secondary reconstruction from the CBCT slices, The view shows the short implants on both sides of the upper jaw. (**B**) clinical photograph for the pre operative condition. (**C**) missing prosthetic part of the implant and adjacent crown showed mobility due to bone loss (poor prognosis) on the right side of the upper jaw. (**D**) the exposed short implants after removing the prosthetic part. (**E**) short implants were removed from the right side of the upper jaw. (**F**) after flap reflection the available bone shows sever loss (**G**) placement of new implants and showing the sinus window; (**H**) Paste-CPC was used as a bone graft to close the sinus window, and filled up the cover the implants and the missing buccal plate in the mesial implant and flap after healing. (**I**) removal of prosthetic part from the left side of the upper jaw. (**J**) Flap opening. (**K**) bone loss is also apparent on the left side. (**L**) placement of new implants on the left side; (**M**) Paste-CPC was used as a bone graft to close the sinus window, and filled up the cover the implants. (**N**) Radiographic image (OPG) represents the inserted long implants and the injected cement. (**O**) suturing of the flap. (**P**) wound healing after 10 days of surgery. (**Q**) Gingival formers were placed for tissue management. (**R**) Impression posts were used for indirect backup impression technique for the upper jaw. (**S**) Screw retained metal framework Try-in on both sides. (**T**) screw retained suprastructure at week 10 post surgery. (**U**) Radiographic view acquired by using 2D basis images for Secondary reconstruction from the CBCT slices, The view showing the inserted implants on the lower jaw after teeth extraction and upper jaw after sinus lifting. (**V**) Impression posts were used for indirect backup impression technique for the lower jaw. (**W**) Screw retained metal framework Try-in for the lower jaw. (**X**) and (**Y**) final result showing the permanent prosthetic parts on the lower and upper jaw, reflecting the resulted finish line and the occlusion.
